# Cancer Cell-Intrinsic PD-1 and Implications in Combinatorial Immunotherapy

**DOI:** 10.3389/fimmu.2018.01774

**Published:** 2018-07-30

**Authors:** Han Yao, Huanbin Wang, Chushu Li, Jing-Yuan Fang, Jie Xu

**Affiliations:** State Key Laboratory for Oncogenes and Related Genes, Division of Gastroenterology and Hepatology, MOH Key Laboratory of Gastroenterology and Hepatology, Renji Hospital, School of Medicine, Shanghai JiaoTong University, Shanghai, China

**Keywords:** tumor cell-intrinsic programmed death 1, combinatorial immunotherapy, mammalian target of rapamycin, tumor growth, blockade

## Abstract

Programmed death 1 (PD-1) and its two natural ligands PD-L1 and PD-L2 are responsible for delivering inhibitory signals that regulate the balance between T cell activation, tolerance, and immunopathology. In previous studies, PD-1 was found only expressed on the surface of immune cells, such as T cells and B cells while PD-1’s ligands PD-L1 and PD-L2 were found expressed in some tumor cells. However, recent studies revealed intrinsic expression of PD-1 in melanoma and some other cancers. In melanoma cells, PD-1 can be activated by its ligand PD-L1 expressed by tumor cells, modulating downstream mammalian target of rapamycin signaling and promoting tumor growth independent of adaptive immunity. In addition to melanoma, PD-1 was also detected in liver cancer cells as well as in non-small lung cancer cells. Unlike its oncogenic functions in melanoma and hepatic carcinoma cells, PD-1 seemed to play a distinct role in lung cancer, as blockade of PD-1 instead promoted tumor cells proliferation. Tumor-intrinsic PD-1 expression seems to be widespread in many tumor types, according to our reanalysis on cancer transcriptomic and proteomic data. The multifaceted roles of PD-1 in tumor cells beyond immune checkpoint signaling may explain the differential therapeutic effects of anti-PD-1 and anti-PD-L1 drugs and provide crucial information when developing combinatorial approaches to enhance antitumor immunity.

## Introduction

As the second generation clinical target of immune checkpoint, programmed death 1 (PD-1) is protein of the CD28 superfamily and a kind of cell membrane protein with 288 amino acids (1). PD-1 is expressed on surface of activated T cells as an inhibitory receptor (2), while its ligands PD-L1 and PD-L2 are mainly expressed in antigen-presenting cells and tumor cells (3, 4). After binding to its ligand, PD-1 suppresses the tumor-killing activity of T cells and downregulates T cells responses. The functions of PD-1 in immune cells include the induction and maintenance of peripheral immune tolerance, protecting tissue from immune attack and dampening infectious immunity and tumor immunity (5). Anti-PD-1 and anti-PD-L1 can relieving the immunosuppressive state of T cells through competitively combining with PD-1 or PD-L1 (6). Because of the relatively satisfactory therapeutic effects, anti-PD-1 drugs, such as nivolumab and pembrolizumab, have been approved by FDA for the treatment of patients with advanced melanoma. However, most patients do not show durable remission, and some tumors have been completely refractory to response with checkpoint blockade, highlighting the requirement of understanding the role of PD-1/PD-L1 axis during the oncogenic and metastatic processes (7–9). In recent studies, the expression of PD-L1 and PD-L2 has been studied in different cancers and stages (10–13). However, most studies on PD-1 expression have focused on immune cells, rendering its potential expression and functions in tumor cells remaining largely unclear. This review summarizes our recent understanding on the multifaceted roles of tumor cell-intrinsic PD-1, aiming to present this interesting research topic to the attentions of researchers in the field of immunotherapy.

## Skin and Liver Cancer Cell-Intrinsic PD-1 Promotes Tumorigenesis

Malignant melanoma is characterized by early metastasis, rapid progression, poor prognosis, and high mortality, and a large number of antibody drugs for malignant melanoma have entered the clinical research stage (14, 15). Especially in 2014, new antibody drugs targeting the PD-L1:PD-1 interaction (pembrolizumab and nivolumab) were approved by FDA for the treatment of metastatic melanoma (7, 9). As in many other cancers, the therapeutic effects of anti-PD-1 drugs in melanoma were thought as a result of enhanced immunity (16, 17). Most previous research of PD-1 were based on its T-cell specific expression, but there have been emerging studies showing its expression and functions in tumor cells including melanoma and hepatoma carcinoma, even in the above tumor-bearing mice lacking adaptive immunity (18–20). Unlike the reported expression of PD-1 in immune-competent cells of the hematopoietic lineage, two independent studies demonstrated that melanoma and liver cancer cell lines and tissue specimens may express the PD-1 protein.

**Figure 1:**
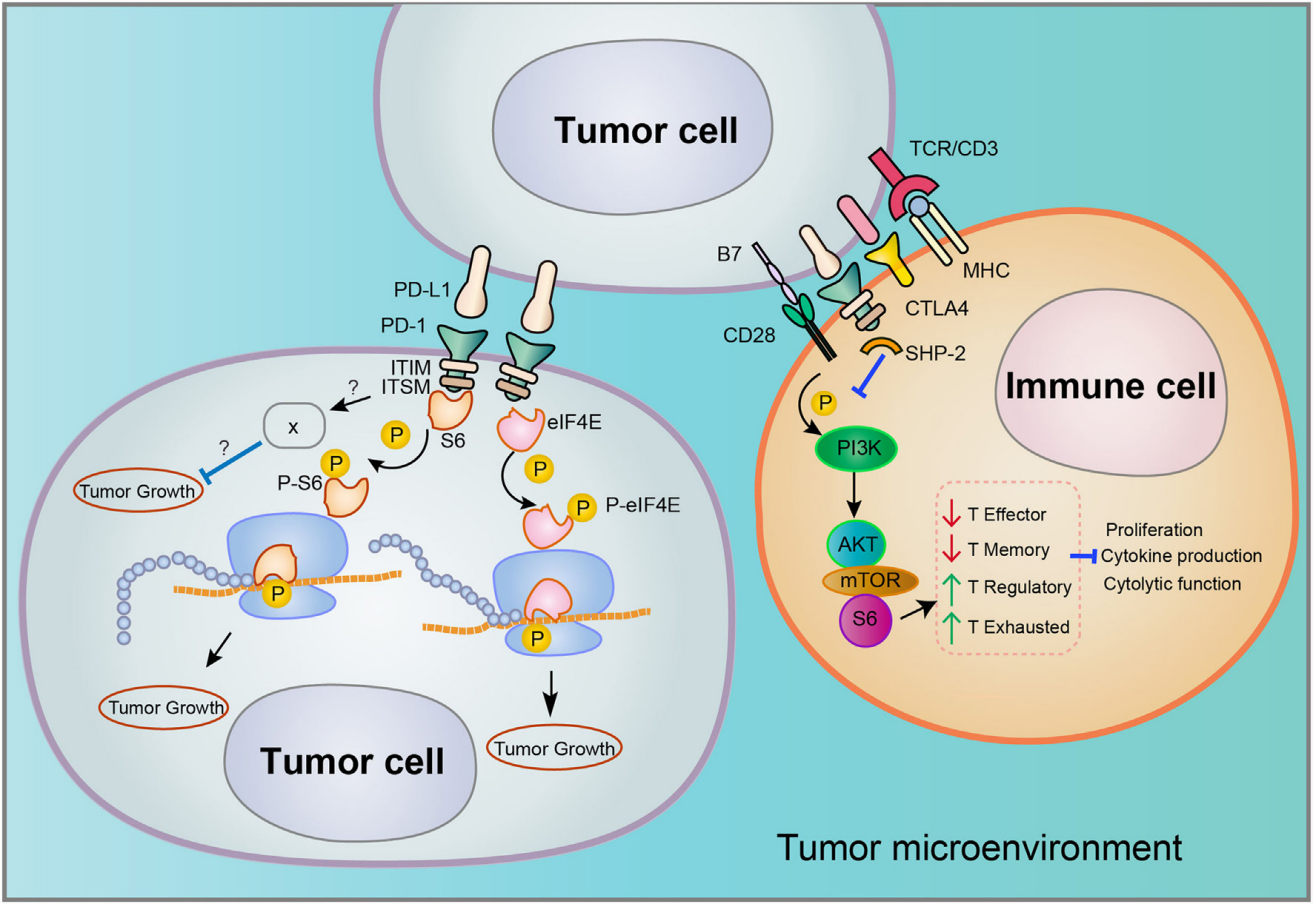
Distinct signaling pathways involved in programmed death 1 (PD-1)/PD-L1 interaction in the tumor microenvironment. Tumor cells may express both PD-L1 and PD-1, but the downstream signaling of PD-1/PD-L1 interaction that occurs in tumor–tumor or tumor–immune cell interfaces may vary considerably. Both the cell type and tumor type may determine the associated signaling pathways.

The biological functions of PD-1 have been intensively studied in T cell, mainly characterized by the binding on cell surface to its ligands PD-L1/PD-L2 and downstream signaling involved in the suppression of T cell proliferation, cytokine production, and cytotoxic functions (schematics in Figure 1) (21). Nevertheless, melanoma or hepatoma carcinoma-intrinsic PD-L1 was found to promote tumor growth even in the absence of functional adaptive immune system. In melanoma cells, PD-1 increased phosphorylation of ribosomal protein S6 (RPS6) as an effector of mammalian target of rapamycin (mTOR) signaling (18). Accordingly, S6 phosphorylation dependent of melanoma-PD-1 could be reversed *via* specific inhibitor of mTOR but not PI3K, demonstrating that PD-1 receptor on surface of melanoma activates downstream mTOR signaling independent of PI3K to promote tumor proliferation (18).

**Figure 2:**
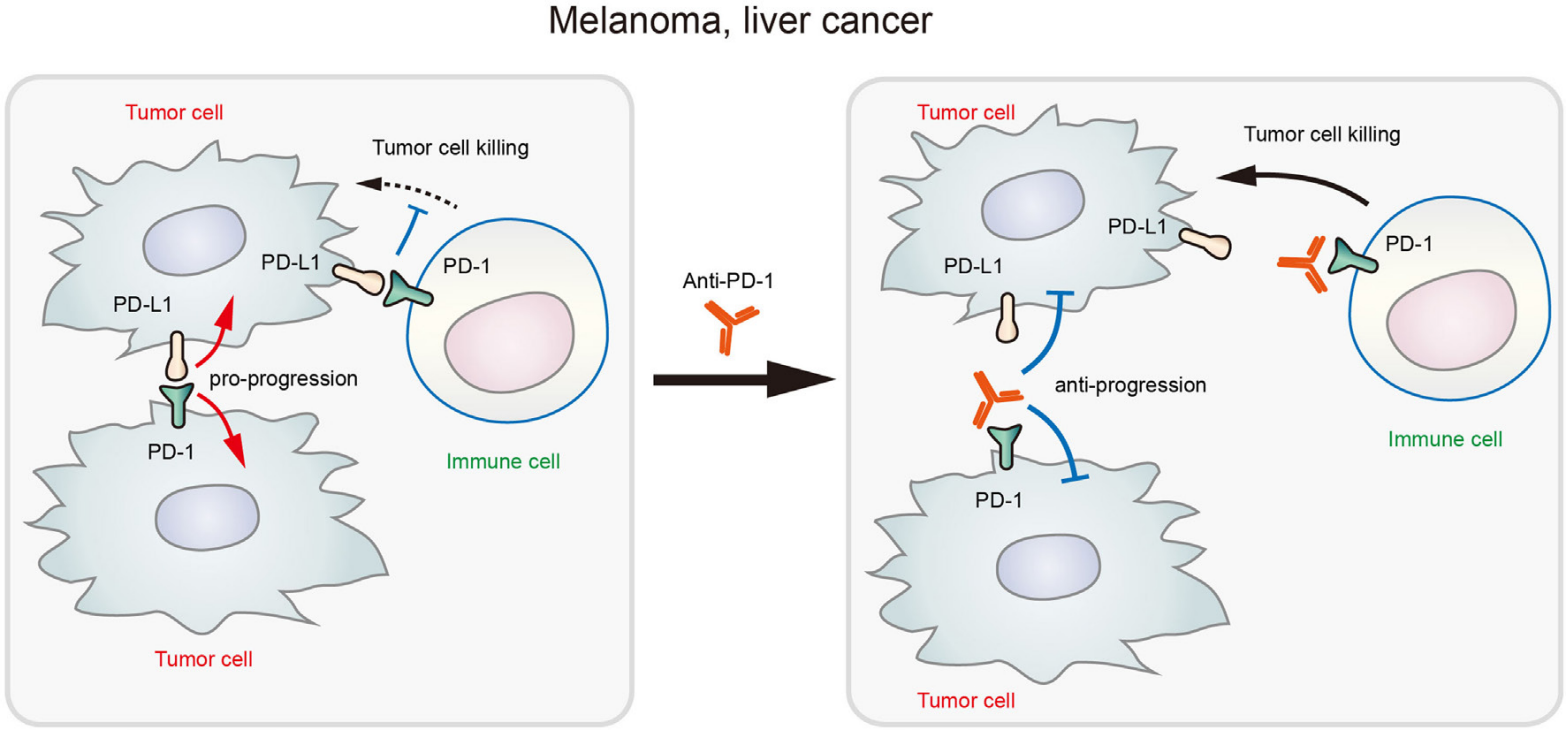
Schematic diagram showing that blockade of tumor-intrinsic programmed death 1 (PD-1) suppresses tumor growth in melanoma and liver cancer.

In addition to melanoma, PD-1 expression was also found in hepatoma carcinoma cells (20). In xenografted and genetically engineered orthotopic HCC models, antibody blockade of PD-1 displayed therapeutic effects by enhancing tumor-killing ability of T cells. Mechanistically, anti-PD-1 reduced the expression of PD-1 on T cells and the binding to its ligands PD-L1 or PD-L2 on hepatoma carcinoma cell, promoting the activation of T cells and cytokine production. However, a recent research showed that both HCC cell lines and clinical HCC tissues may contain subpopulations that express PD-1, and HCC cell-intrinsic PD-1 promotes tumor progression even in the absence of an immunological environment [20]. By contrast, PD-1 blockade and knockdown (KD) *in vitro* and *in vivo* inhibited tumor growth independently of adaptive immunity. In these tumor cells, the cytosolic domains of PD-1 was found to interact with the eukaryotic initiation factor 4E and RPS6, promoting the phosphorylation of these mTOR effector proteins (Figure 1) (20). The authors also proposed that anti-PD-1 drug may function by both stimulating antitumor immunity and blocking the pro-tumorigenic functions of tumor-intrinsic PD-1 (schematics in Figure 2). In support to this proposed model, combination of mTOR inhibitors and PD-1 antibody provided more durable and synergistic tumor regression than either single agent alone, each of which presented only modest efficacy.

## Blockade of PD-1 in Non-Small Cell Lung Cancer Promoted Tumor Growth

**Figure 3:**
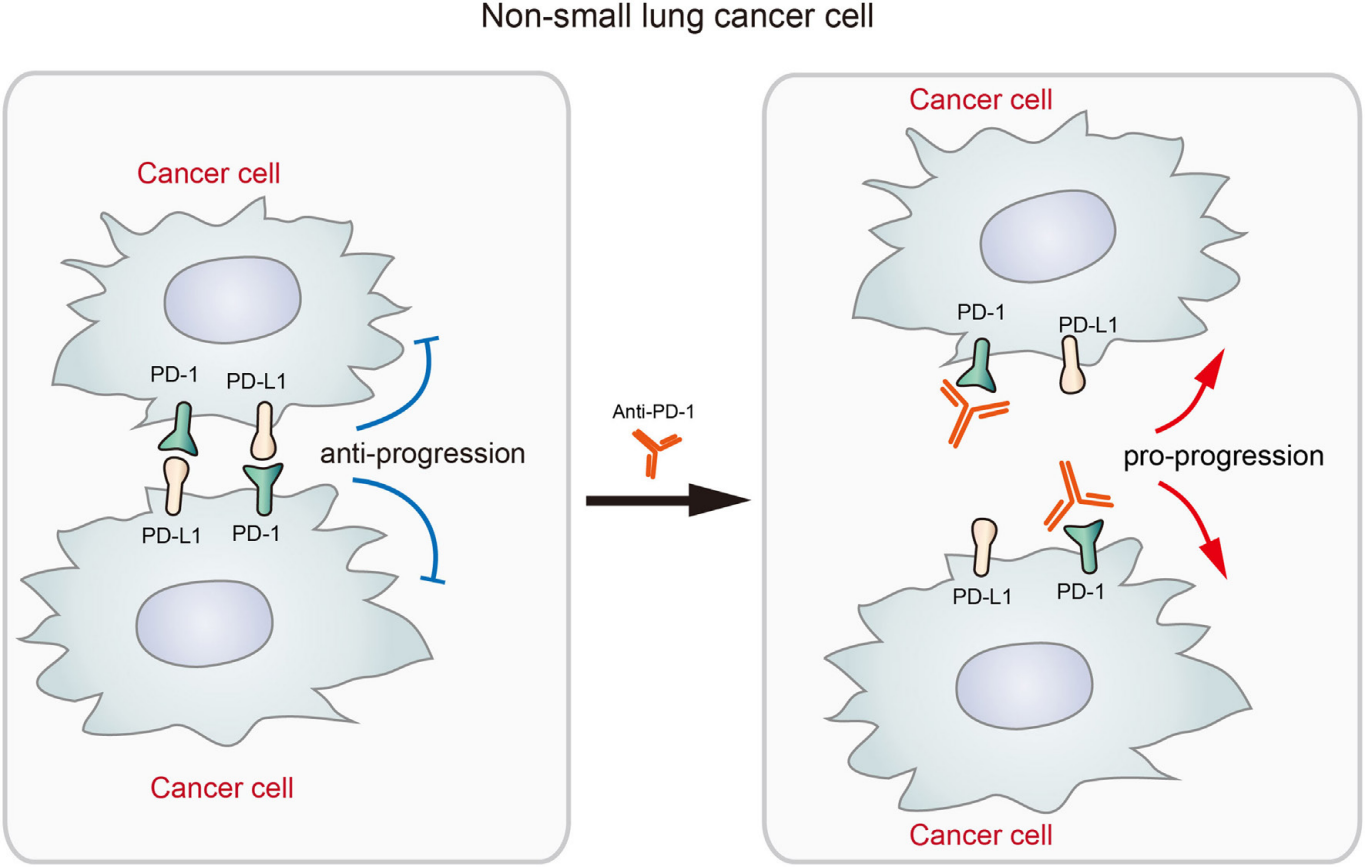
The diagram shows that the blockade of tumor-intrinsic programmed death 1 (PD-1) promotes tumor growth in lung cancer. The interaction between tumor-intrinsic PD-1 and PD-L1 inhibits tumor progression, but the treatment by anti-PD-1 disrupts this inhibitory signaling and promotes tumor progression. This process represents an adverse effect of anti-PD-1 therapy for activating antitumor immunity.

Given the significant therapeutic effects displayed by checkpoint blockade therapy, FDA approved several anti-PD-L1 or anti-PD-1 drugs in the treatment of advanced cancers including non-small cell lung cancer (NSCLC) (22, 23). Although many NSCLCs displayed durable response to anti-PD-1 or anti-PD-L1 drugs (7), a recent study by Du et al. reported the expression of PD-1 in NSCLC cells and its potential adverse effects to checkpoint blockade therapy (19). In an NSCLC patient expressing tumor-intrinsic PD-1, the tumor rapidly progressed upon anti-PD-1 therapy for 2 months. In the M109 murine NSCLC cell line, PD-1 overexpression significantly decreased cell viability while PD-1 KD increased cell viability (19). The results suggested that blockade of NSCLC-intrinsic PD-1 released PD-1 from tumor-suppression effects under its interaction with the ligands, promoting the growth of NSCLC (described in Figure 3). Although the exact mechanisms remain unclear, the author speculated that it may be due to the complex consequences by the phosphatases that interact with activated PD-1 (19). It suggested the possibility that anti-PD-1 immunotherapy will be rendered less efficacious or even deleterious in some patient and it is very necessary to elucidate the mechanism how cell-intrinsic PD-1 regulates tumor growth and development in different tumors.

## Implications in Refining Combinatorial Immunotherapies

Cytotoxic T lymphocyte associated antigen-4 (CTLA-4) is the clinical target of the first generation of immune checkpoint (24). In March 2011, ipilimumab (anti-CTLA-4) was approved by FDA for treatment of advanced melanoma (25). PD-1 is the clinical target of the second generation of immune checkpoint (26), and then both nivolumab and pembrolizumab have been approved by FDA for treatment of malignant melanoma (7, 9). The existence of tumor cell-intrinsic PD-1 and its tumor-regulatory effects may explain some baffling question as follows. In addition to tumors with high immunogenicity, PD-1 blockade has also displayed clinical activity in patients with less immunogenic cancers, which have no obvious response to immunotherapy targeting other immune checkpoints (27–30). For instance, patients with advanced melanoma refractory to treatment with ipilimumab, which is targeting CTLA-4, showed good clinical response to anti-PD-1 therapy (27, 30). It should be noted that the presence of neoantigens and immune-active microenvironment of patients with treatment of PD-1 blockade are similar with patients with CTLA-4-directed checkpoint blockade (31, 32). Moreover, Postow et al. found that PD-1 inhibitors produced greater anticancer activity and fewer immune-related adverse events compared with anti-CTLA-4, ipilimumab (7). These observations collectively suggested that PD-1 antibody may target not only T-cell-specific immune checkpoint functions but also some pro-tumorigenic mechanisms. Since PD-1 is expressed not only on immune cells but also on tumor cells, the superior clinical activity and safety profile of anti-PD-1 compared with anti-CTLA-4 therapy may be due to the additional effects of anti-PD-1 on targeting tumor-intrinsic signaling (7, 9, 28). However, in this context, the principle of precision medicine seems to be crucial, because the roles of tumor-intrinsic PD-1 seem to vary considerably in different tumor types. As PD-1 blockade may promote tumor growth in NSCLC and possibly other cancers, it may be plausible to apply anti-PD-L1 instead of anti-PD-1 for checkpoint blockade, or to combine antiproliferative drugs with anti-PD-1 therapy. In summary, further clarifying the roles of tumor-intrinsic PD-1 may create vast opportunity for refining combinatorial immunotherapy, and extensive efforts are required to precisely define the roles of PD-1 in different cancer types, cell types, and individual cancer cases.

## Expression of PD-1 in Different Cancer Tissues and Cells

**Figure 4:**
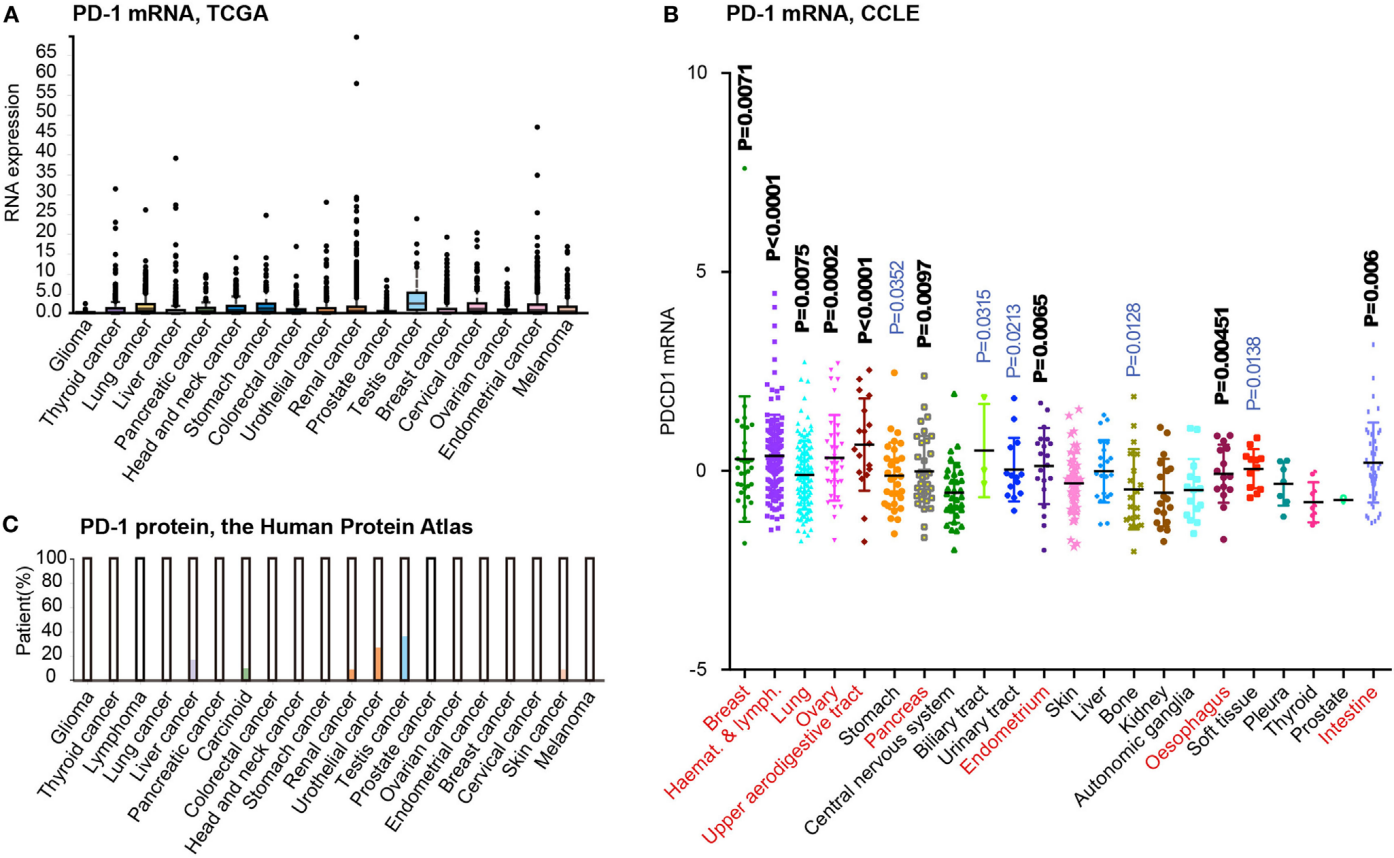
Tumor-intrinsic programmed death 1 (PD-1) expression is widespread in many tumor types. **(A)** PD-1 mRNA expression level analysis in different cancer tissues from the human protein atlas. **(B)** PD-1 mRNA expression level analysis in various kinds of cancer cells based on related data from Cancer Cell Line Encyclopedia (CCLE). **(C)**. PD-1 protein expression level analysis in different cancer tissues using immunohistochemical staining from the human protein atlas. All cancer types were compared to “Central nervous system” tumors by t-test (P-values indicated). Tumors displaying highly significant differences (*P*<0.01) are marked by red font.

Recent advances in cancer genomic studies have enabled the reanalysis of PD-1 expression in different cancer types. By obtaining and preprocessing the mRNA expression data from The Cancer Genomic Atlas (TCGA) project (33), we have summarized the expression of PD-1 in the tissues of 17 cancer types (Figure 4A). Interestingly, most tumor types contain a number of cases expressing higher levels of PD-1 mRNA (above the SD as shown in the box plot). However, considering that cancer tissues may contain infiltrating immune cells, the microarray data from TCGA may not accurately reflect the PD-1 mRNA level in tumor cells. Thus, we reanalyzed the expression of PD-1 mRNA in Cancer Cell Line Encyclopedia (CCLE) dataset, which was based on cultured cancer cell lines and thus without the suspect of immune cell contamination. This analysis including 22 tumor types and 617 cell lines revealed unified low expression of PD-1 in only a few cancer types such as glioma (central nervous system), thyroid cancer, and prostate cancer (Figure 4B). But comparing to these tumors, most tumor types exhibited higher expression levels and greater variability in PD-1 expression (*P* < 0.001, *t*-test, highlighted in red font in Figure 4). To further analyze the PD-1 protein expression in different cancers, we also summarized the immunohistochemical (IHC) staining results in the Human Protein Atlas (34). Although the IHC results may be affected by more factors (e.g., the specificity/sensitivity of the antibody, the view field selected for analysis, and the quantification approaches, etc.), this approach enabled selective analysis on the expression of PD-1 protein tumor cells. The results suggested that liver cancer, carcinoid, renal cancer, urothelial cancer, testis cancer, and melanoma (skin cancer) have subgroups of tumor cells with positive PD-1 staining (Figure 4C). The driving force of PD-1 expression in tumor cells is unknown, but we speculate that multiple factors may be involved, such as gene copy number alterations, epigenetic alterations, and microenvironment, etc. According to the CCLE dataset, PD-1 is amplified in some tumor cell lines, with potential effects on PD-1 mRNA expression. In addition, the cytokines and immune cells in the tumor microenvironment may also participate in the induction of tumor PD-1 expression. Although there is yet no evidence that PD-1 may be transferred from leukocytes to tumor cells, it certain deserves in-depth studies in the future.

Taken together, the analysis of PD-1 expression at both mRNA and protein levels prompted us that PD-1 expression may be more prevalent than the reported three tumor types. Therefore, understanding the roles of cancer cell-intrinsic PD-1 may have broad implications in the refinement of combinatorial immunotherapies.

## Conclusion

Although the roles of PD-1 in leukocytes have been well established, the expression of PD-1 in tumor cells has been less characterized, with the potential functions largely unclear. When the expression of PD-1 on melanoma cells was reported in 2015, it was not considered as a widespread mechanism. But after the recent studies on the expression and roles of PD-1 in liver cancer and lung cancer, the prevalence and functional importance of tumor-intrinsic PD-1 has attracted the attentions of more researchers. Although accumulating evidence suggests that mTOR signaling may play a role in this scenario, our understanding on the biological roles and therapeutic implications of tumor-intrinsic PD-1 remain very limited. Future in-depth investigations on tumor-intrinsic PD-1 may provide additional insights into the unexpected effects of checkpoint blockade therapies and benefit the development of more effective combinatory immunotherapies.

## Author Contributions

HY and JX wrote the manuscript, generated the schematics and analyzed data. HW, CL and JYF contributed to the revision of the manuscript.

## Conflict of Interest Statement

The authors declare that the research was conducted in the absence of any commercial or financial relationships that could be construed as a potential conflict of interest.
